# Agricultural management and cultivation period alter soil enzymatic activity and bacterial diversity in litchi (*Litchi chinensis* Sonn.) orchards

**DOI:** 10.1186/s40529-021-00322-9

**Published:** 2021-09-26

**Authors:** Yu-Pei Chen, Chia-Fang Tsai, Asif Hameed, Yu-Jen Chang, Chiu-Chung Young

**Affiliations:** 1 Department of Public Health and Medical Technology, Xiamen Medical College, Xiamen, 361023 Fujian China; 2 Engineering Research Center of Natural Cosmeceuticals College of Fujian Province, Xiamen Medical College, Xiamen, 361023 Fujian China; 3 Department of Soil and Environmental Sciences, National Chung Hsing University, Taichung, 40227 Taiwan; 4 Bioresource Collection and Research Center, Food Industry Research and Development Institute, Hsinchu, 300 Taiwan; 5 Innovation and Development Center of Sustainable Agriculture, National Chung Hsing University, Taichung, 40227 Taiwan

**Keywords:** Agricultural management, Temporal change, Soil enzymes, Bacterial community, Bacterial diversity

## Abstract

**Background:**

Agricultural management and temporal change including climate conditions and soil properties can result in the alteration of soil enzymatic activity and bacterial community, respectively. Therefore, different agricultural practices have been used globally to explore the soil quality. In this study, the temporal variations in soil property, enzymatic activity, and bacterial community at three successive trimester sampling intervals were performed in the soil samples of litchi orchards that were maintained under conventional and sustainable agricultural practices.

**Results:**

Agricultural management found to significantly influence arylsulfatase, β-glucosidase, and urease activities across time as observed by repeated-measures analysis of variance. Shannon and Simpson diversity indices, and the relative abundance of predominant Acidobacteria and Proteobacteria were significantly influenced by temporal change but not agricultural management. This suggested that soil enzymatic activity was more susceptible to the interaction of temporal change and agricultural management than that of the bacterial community. Multiple regression analysis identified total nitrogen, EC, and phosphorus as the significant predictors of acid phosphatase, arylsulfatase, and β-glucosidase for explaining 29.5–39% of the variation. Moreover, the soil pH and EC were selected for the SOBS, Chao, ACE, and Shannon index to describe 33.8%, 79% of the variation, but no significant predictor was observed in the dominant bacterial phyla. Additionally, the temporal change involved in the soil properties had a greater effect on bacterial richness and diversity, and enzymatic activity than that of the dominant phyla of bacteria.

**Conclusions:**

A long-term sustainable agriculture in litchi orchards would also decrease soil pH and phosphorus, resulting in low β-glucosidase and urease activity, bacterial richness, and diversity. Nevertheless, application of chemical fertilizer could facilitate the soil acidification and lead to adverse effects on soil quality. The relationship between bacterial structure and biologically-driven ecological processes can be explored by the cross-over analysis of enzymatic activity, soil properties and bacterial composition.

**Supplementary Information:**

The online version contains supplementary material available at 10.1186/s40529-021-00322-9.

## Background

Soil enzymatic activities and microorganisms involved in soil biochemical processes have been suggested as indicators of soil quality. Nevertheless, soil enzymes are sensitive to certain environments and conditions in soil due to the multivariate turn-over times of enzymes (Schimel et al. 2017). In contaminated soils, β-glucosidase associated with the carbon cycle could be a biological indicator of soil health (Dai et al. 2018). This was also reflected in the arylsulfatase activity of heavy metal contaminated soils (Xian et al. 2015). Moreover, soil enzyme activity can directly inspect the transformation and fertility level of soil elements and nutrients. An overall increasing trend of soil enzymatic activity was observed with long-term rice cultivation in mudflat soil, attributed to salt desalination and organic matter accumulation (Zhang et al. 2019). Thus, soil enzymes are imperative in the evaluation of the ecological health of the terrestrial ecosystem.

On the other hand, much attention has been devoted to the relationship between soil microbial abundance and diversity, and soil properties (Bakker et al. 2018; Murphy et al. 2016). Several environmental factors including agricultural management, soil pH, organic carbon, nitrogen, and temporal change can shift the soil microbial population (Kivlin and Hawkes 2016; Shigyo et al. 2019). A considerable variation in microbial community structure was observed between organically and conventionally managed soils because of the differences in the nutrient inputs and agronomic practices (Arcand et al. 2017; Bakker et al. 2018; Chen et al. 2018). Microbial carbon use efficiency can be affected by microbial communities through altering agricultural management (Arcand et al. 2017). This results from the crop residue decomposition between the organically and conventionally agricultural practices. Besides, soil pH was assumed to be the main driver for the prokaryotic community structure (Fierer and Jackson 2006; Lammel et al. 2018; Lauber et al. 2009; Shen et al. 2019). It can directly affect the enzymatic activity and bacterial composition or indirectly influence the solubility of different elements such as phosphorus, aluminum, iron, copper, molybdenum, and zinc (Lammel et al. 2018).

The effect of temporal dynamic including climate conditions and soil properties on soil enzymatic activity and microbial community is important for improving ecosystem management (Armstrong et al. 2016; Park et al. 2015; Shigyo et al. 2019). Several studies have been published on a low bacterial abundance in the summer (Jung et al. 2012; Rasche et al. 2011). The contribution of seasonal changes to microbial communities has a greater effect than that of spatial heterogeneity in cool-temperature montane forests (Shigyo et al. 2019). Additionally, temporal variability involved in temperature change can affect the soil spinach rhizosphere microbial community (Mark Ibekwe et al. 2017). Kivlin and Hawkes’ study indicated that bacterial richness and diversity were influenced by the interaction of vegetation type and time (Kivlin and Hawkes 2016).

Litchi (*Litchi chinensis* Sonn.) is a tropical fruit with high commercial value cultivated in tropical and subtropical regions (Ray 2002). Fruit and seeds of litchi reportedly exhibit anticancer, antibacterial and antiviral features (Ibrahim and Mohamed 2015). According to conventional management, clean tillage can control the understory weed to prevent the competition of nutrients between fruits and weed; however, this can result in deterioration of the soil structure and lead to soil erosion and nutrient loss (Liu et al. 2013). Furthermore, the long-term application of chemical fertilizer has been demonstrated to cause a reduction in biological activities, loss of soil organic matter, and destroying soil structure (Bronick and Lal 2005; Zhao et al. 2016). Therefore, sustainable management with mixed cropping, cover crops, and rational fertilization has been widely accepted. No attention has been focused on the relationship between the bacterial community and enzymatic activity under conventional and sustainable management in the litchi orchards. Moreover, the temporal change in environmental factors shaping soil bacterial structure and enzymatic activity remains poorly understood. Thus, the two subjects were addressed that (i) which enzymatic activities can be affected by temporal changes and agricultural management practices; and (ii) which soil properties can mainly influence enzymatic activities and bacterial community. In this study, two litchi orchards under conventional and sustainable management were compared by determining the activities of different enzymes and soil bacterial communities in the time course. The cross-over analysis can further examine how the soil enzymatic activity and bacterial composition alter under different agricultural management with temporal change.

## Materials and methods

### Field site and soil chemical analyses

The experimental field was located in the Taiwan Agricultural Research Institute, Council of Agriculture, Chiayi Agricultural Research Branch (23° 29′ 5.1288″ N, 120° 28′ 12.2592″ E) (Additional file 1: Fig. S1). The litchi orchards (*L. chinensis* Sonn.) were managed by conventional (CA; 1.32 ha), and sustainable agriculture (SA; 1.33 ha) since 2006. The soils were classified as Hapludults according to the soil taxonomy of the United States Department of Agriculture. The litchi orchards performed by the conventional and sustainable agriculture had been planted for over 15 and 70 years, respectively. In the SA litchi orchard, no herbicide, pesticide, organic fertilizer, and chemical fertilizer were applied before 2007. Between 2007 and 2017, the soil was manually weeded without fertilizer application, and litchi had been slightly treated with herbicide in the SA. Moreover, litchi fruit was not harvested and dropped off naturally. CA soil was annually carried out with field irrigation, organic fertilizer, chemical fertilizer, herbicide, and pesticide. Additionally, the CA soil was also weeded, and the litchi tree was trimmed (Additional file 3: Table S1). The fruit in the CA litchi orchard was harvested from May to June every year. The temperatures and relative humidity at the sampling times, October 4, 2016 (CA1 and SA1), January 4, 2017 (CA2 and SA2), and April 11, 2017 (CA3 and SA3), were 29.8 °C, 23.5 °C, 29.9 °C, and 72%, 70%, and 53%, respectively. The CA and SA soils respectively have three and five sampling sites because of the division of planting areas and road distinction. The surface soils (depth 0–10 cm) from five plots of each sampling site were collected and pooled with replicate in sterile plastic tubes. To determine the enzymatic activity, the soil samples were directly brought back to the laboratory, sieved by using a 2-mm mesh, and homogenized by using a centrifugal ball mill (S100, Retsch, Hahn, Germany). The other soil samples stored at − 80 °C were used for DNA extraction and chemical analysis. Soil suspension prepared in distilled water (1:1, w/v) was used for testing pH and electrical conductivity. Organic matter was determined by a loss on the ignition after burning at 430 °C for 24 h. The Kjeldahl method was used to measure total nitrogen. The soil extractable elements including P, K, Ca, Mg, Fe, Mn, Cu, and Zn were determined by inductively coupled plasma (ICP) emission spectroscopy (Hendershot et al. 2008).

### Soil enzymatic activity

*p*-Nitrophenyl phosphate, *p*-nitrophenyl sulfate, and *p*-nitrophenyl-*β*-d-glucopyranoside were respectively prepared as substrates for determining the enzymatic activities of acid phosphatase, arylsulfatase, and *β*-glucosidase (Eivazi and Tabatabai 1988; Ho 1979; Tabatabai and Bremner 1970). To measure the acid phosphatase, 0.5 g of soil was added into a mixture of 0.5 mL of 115 mM *p*-nitrophenyl phosphate, 0.2 mL of toluene, and 2 mL of modified universal buffer (MUB), and further incubated at 37 °C for 1 h. According to the Tabatabai method (Tabatabai 1982), MUB was manufactured including Tris, maleic acid, citric acid, boric acid, NaOH, HCl, and distilled water at pH 6.5. For arylsulfatase and *β*-glucosidase activity assay, the composition of the reaction was the same as that of acid phosphatase. Nevertheless, the substrate was replaced by 25 mM *p*-nitrophenyl sulfate and 25 mM *p*-nitrophenyl-*β*-d-glucopyranoside, respectively. These mixtures were incubated at 37 °C for 1 h. Then, 2 mL of 0.5 M NaOH and 0.5 mL of 0.5 M CaCl_2_ were respectively added into the mixture to cease the enzymatic reaction of acid phosphatase and arylsulfatase. To terminate the *β*-glucosidase activity, 2 mL of 0.5 M Tris buffer (pH 12) and 0.5 mL of 0.5 M CaCl_2_ were added. These mixtures were filtered by using filter paper (No. 393, Sartorius AG, Goettingen, Germany) and measured at OD_400_ by a spectrophotometer. Different concentrations of *p*-nitrophenol were utilized as the standard to calculate the enzymatic activity. Urease activity was determined according to Kandeler and Gerber (1988). One gram of soil was added into 0.5 mL of 0.08 M urea and incubated at 37 °C for 2 h. Then, 10 mL of 1 N KCl was added into the mixture to stop the reaction and filtered (No. 393, Sartorius AG, Goettingen, Germany) to remove debris. The 400 µL of 0.1% dichloroisocyanuric acid sodium salt, 1 mL of sodium salicylate (Na-salicylate/NaOH), and 1.8 mL of distilled water were mixed with 200 µL filtrate, and further incubated in the dark for 30 min. The reaction was determined at OD_690_ by using a spectrophotometer. Different concentrations of NH_4_Cl were utilized as the standard to calculate the enzymatic activity.

To measure the N_2_-fixing activity of soils, the acetylene reduction method was utilized (Hardy et al. 1973). An 80-mL tube filled with 17 mL of soil and a 10% acetylene gas mixture was incubated at 25 °C for 24 h. A gas chromatography (GC) instrument (HITACHI model 163, Hitachi Ltd., Tokyo, Japan) equipped with a flame ionization detector (FID) and a packed column (1.0 m × 2.0 mm internal diameter, steel column packed with Porapak-T 80–100) was used to determine the ethylene catalyzed by soil nitrogenase. The GC parameters were as follows: carrier gas, nitrogen; flow rate, 35 mL h^− 1^; FID temperature, 110 °C; and column temperature, 65 °C.

### Soil bacterial 16S rDNA amplicon sequencing by Illumina MiSeq and bioinformatic analysis

The soil bacterial distribution was explored through soil DNA sequencing analysis using next-generation sequencing. The UltraClean Soil DNA Isolation Kit (MO BIO Laboratories, Inc., USA) with 1-g soil was used to extract soil DNA detected by a spectrophotometer at OD260/280 ratio in a range of 1.8–2.0. The primers of 338F (5′-ACTCCTACGGGAGGCAGCAG-3′) and 806R (5′-GGACTACHVGGGTWTCTAAT-3′) for the hypervariable V3-V4 region of the 16S rRNA were used for PCR (Caporaso et al. 2012; Lane 1991). The DNA sample using 30 ng was amplified by PCR, containing 0.75 U PrimeSTAR HS DNA polymerase (Takara Bio, Japan), 1 × PrimSTAR buffer, 0.2 mM dNTPs, and 10 pM forward and reverse primers. The PCR reaction was performed as follows: 3 min at 94 °C for 1 cycle, followed by 30 cycles of 30 s at 94 °C, 45 s at 55 °C and 45 s at 72 °C, and 10 min at 72 °C for 1 cycle. PCR products were blunted, and adapter ligation was conducted according to the Beijing Genomics Institute (BGI) experimental workflow (Ju et al. 2017; Li et al. 2020; Qin et al. 2012). Moreover, library construction, and amplicon sequencing were entrusted to the BGI (BGI, Shenzhen, China). The all standard protocols were described in the literature provided by BGI-Shenzhen (Li et al. 2020; Qin et al. 2012). The quantification and qualification of the DNA library were detected by the Agilent 2100 bioanalyzer instrument (Santa Clara, CA, USA) and real-time quantitative PCR. The Illumina MiSeq platform (2 × 300 bp paired-end reads) was used for amplicon sequencing.

The raw data were analyzed following the BGI bioinformatics workflow. The consensus sequence with the overlapped two paired-reads was generated by FLASH and merged to tags (Magoc and Salzberg 2011). The tags were clustered to operational taxonomic unit (OTU) with a 97% threshold by USEARCH and UPARSE (Edgar 2013). Chimeras were filtered out by using UCHIME and all tags were mapped to each OTU by using USEARCH GLOBAL (Edgar et al. 2011). The OTU representative sequence for the taxonomic ranks of microbes at 97% similarity was classified using the Ribosomal Database Project (RDP) classifier (Cole et al. 2014) and the Greengenes database (v201305) (DeSantis et al. 2006) was used to identify soil bacterial taxa. The Venn diagrams grouped the OTU representative sequence, the taxonomic distribution of microbes, and the redundancy analysis (RDA) of the relationship between the soil bacteria, enzymatic activities, and chemical properties were created by the R software (v3.1.1) (Foundation for Statistical Computing, Vienna, Austria). Alpha diversity indices including species observed (SOBS), Chao, ACE, Shannon, and Simpson were calculated by Mothur (v1.31.2). SOBS, Chao, and ACE can reflect the soil bacterial richness of community. The rarefaction curve could be used to estimate that the data is enough to cover all species in the soil community according to the three indices. Shannon and Simpson indicated the bacterial diversity of the community, affected by bacterial richness and evenness. These indices of calculation formula can refer to http://www.mothur.org/wiki/Calculators. The samples were grouped based on the sampling time and agricultural management for differential analysis of alpha diversity. The box plot of alpha diversity was drawn by the R software. Beta diversity with weighted UniFrac was done by QIIME (v1.80). The heat map of beta diversity was generated by the package NMF of R software.

### Statistical analysis

The variability among replicates was analyzed by the standard deviation (SD). Statistical significance at a confidence level of 95% was measured through Duncan’s multiple range test by using IBM SPSS Statistics v20 software package (SPSS Inc. Chicago, USA). Repeated measures analysis of variance (RM-ANOVA) was used to examine the interaction of temporal change and agricultural management affected by enzymatic activity, bacterial distribution, and alpha diversity. The RDA implemented in the vegan package of R software was used to analyze the relationship between the soil properties, enzymatic activities, bacterial distribution, and alpha diversity. The difference in soil enzymatic activities, soil bacteria, and soil properties was explored by ANOVA with multivariate linear regression. The responses of enzymatic activity and bacterial community to variance in soil properties were interpreted by the stepwise method (probability use of F < 0.05).

### Nucleotide sequence accession number

This soil high-throughput DNA sequencing project was deposited at DDBJ/ENA/GenBank under accession numbers BioProject PRJNA609234 and BioSample SAMN14239000, SAMN14239003, SAMN14239006, SAMN14239008, SAMN14239071, SAMN14239231, SAMN14239232, SAMN14239233, SAMN14239279, SAMN14247548, SAMN14247549, SAMN14247570, SAMN14247586, SAMN14247633, SAMN14247636, SAMN14247637, SAMN14247688, SAMN14247698, SAMN14247701, SAMN14247707, SAMN14247714, SAMN14247780, SAMN14247782 and SAMN14247783.

## Results

### Soil properties in the litchi orchards

**Fig. 1:**
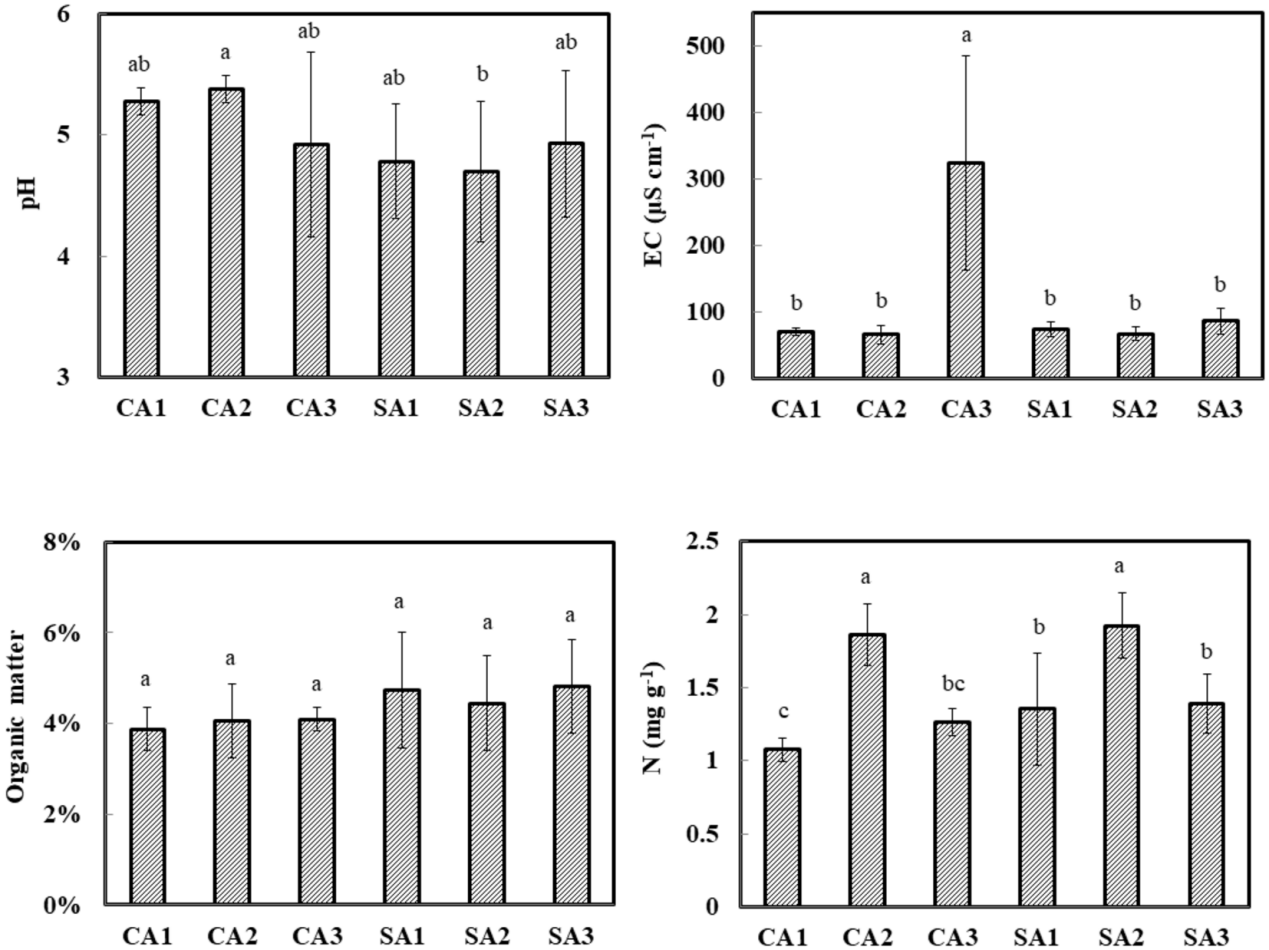
Temporal variations in the soil pH, EC, total nitrogen, and organic matter of litchi orchards that were maintained under conventional (CA) and sustainable (SA) agricultural practices. 1, 2 and 3 indicate successive trimester samplings carried out on October 2016, January 2017 and April 2017, respectively. Results are shown as mean ± standard deviation. The different letters labeled at columns are significantly different according to Duncan’s test (p < 0.05) by IBM SPSS Statistics v20

The soil samples of litchi orchards that were maintained under conventional (CA) and sustainable (SA) agriculture were collected trimester-wise successively from October 2016 and April 2017 to observe the impact of agricultural management and temporal change. The soil pH was ranged from 4.70 to 5.37 in the litchi orchards (Fig. 1). The pH of SA soil was slightly lower than that of CA soil. No obvious change between the EC values of CA and SA soils was observed in October 2016 and January 2017. However, a high soil EC value was detected in the CA soil of April 2017 due to the application of fertilizer in March 2017. The soil organic matter on average was 4.0% and 4.7% in the CA and SA soils, respectively, but there was no significant difference. The result of total nitrogen indicated that the content in the SA soil was slightly higher than in the CA soil. In addition, the total nitrogen was the highest in January 2017, reaching 1.8 mg g^− 1^ soil in both soils. No significant difference was perceived between the CA and SA soils in the extractable elements including Ca, Mg, Mn, and Cu (Table 1). The contents of P and Zn in the CA soil were higher than that in the SA soil. By contrast, the Fe trend was SA > CA. Moreover, the extractable element of K was the highest in the CA soil of April 2017. According to the Pearson correlation analysis of soil properties, soil pH had a high relationship with the extractable elements (Additional file 4: Table S2). A positive correlation between organic matter and total nitrogen, and EC and potassium content was also documented.

**Table 1:** Temporal variations in the soil extractable elements of litchi orchards that were maintained under conventional (CA) and sustainable (SA) agricultural practices

Soils	Elements							
	P	K	Ca	Mg	Fe	Mn	Cu	Zn
	mg kg^− 1^ soil							
CA1	2.3 ± 1.0^bc^	211.0 ± 54.7^b^	583.3 ± 143.1^a^	160.3 ± 18.5^a^	453.3 ± 79.0^a^	104.3 ± 47.5^a^	4.0 ± 2.1^a^	3.5 ± 0.5^b^
SA1	1.9 ± 0.7^c^	145.1 ± 52.3^b^	703.5 ± 371.1^a^	195.9 ± 70.5^a^	428.1 ± 15.3^ab^	114.5 ± 38.1^a^	2.9 ± 1.5^a^	4.0 ± 1.3^b^
CA2	4.3 ± 1.2^ab^	251.3 ± 88.2^b^	882.6 ± 292.8^a^	174.1 ± 29.9^a^	419.2 ± 59.7^ab^	77.0 ± 42.5^a^	5.7 ± 1.8^a^	7.0 ± 1.0^a^
SA2	3.4 ± 1.2^abc^	139.0 ± 47.1^b^	614.9 ± 371.0^a^	160.7 ± 73.3^a^	471.1 ± 21.1^a^	73.0 ± 22.8^a^	4.3 ± 1.9^a^	4.9 ± 2.2^ab^
CA3	4.7 ± 1.5^a^	543.0 ± 504.3^a^	911.5 ± 411.2^a^	149.5 ± 16.7^a^	378.2 ± 64.6^b^	84.2 ± 22.5^a^	5.1 ± 1.9^a^	7.0 ± 0.7^a^
SA3	4.0 ± 1.7^ab^	138.3 ± 39.6^b^	633.3 ± 387.9^a^	144.6 ± 67.5^a^	420.5 ± 10.7^ab^	99.5 ± 47.8^a^	4.4 ± 2.2^a^	5.5 ± 2.4^ab^

1, 2 and 3 indicate successive trimester samplings carried out on October 2016, January 2017 and April 2017, respectively. The extractable elements including P, K, Ca, Mg, Fe, Mn, Cu, and Zn were determined by inductively coupled plasma emission spectroscopy. Results are shown as mean ± standard deviation

### Soil enzymatic activities in the litchi orchards

**Fig. 2:**
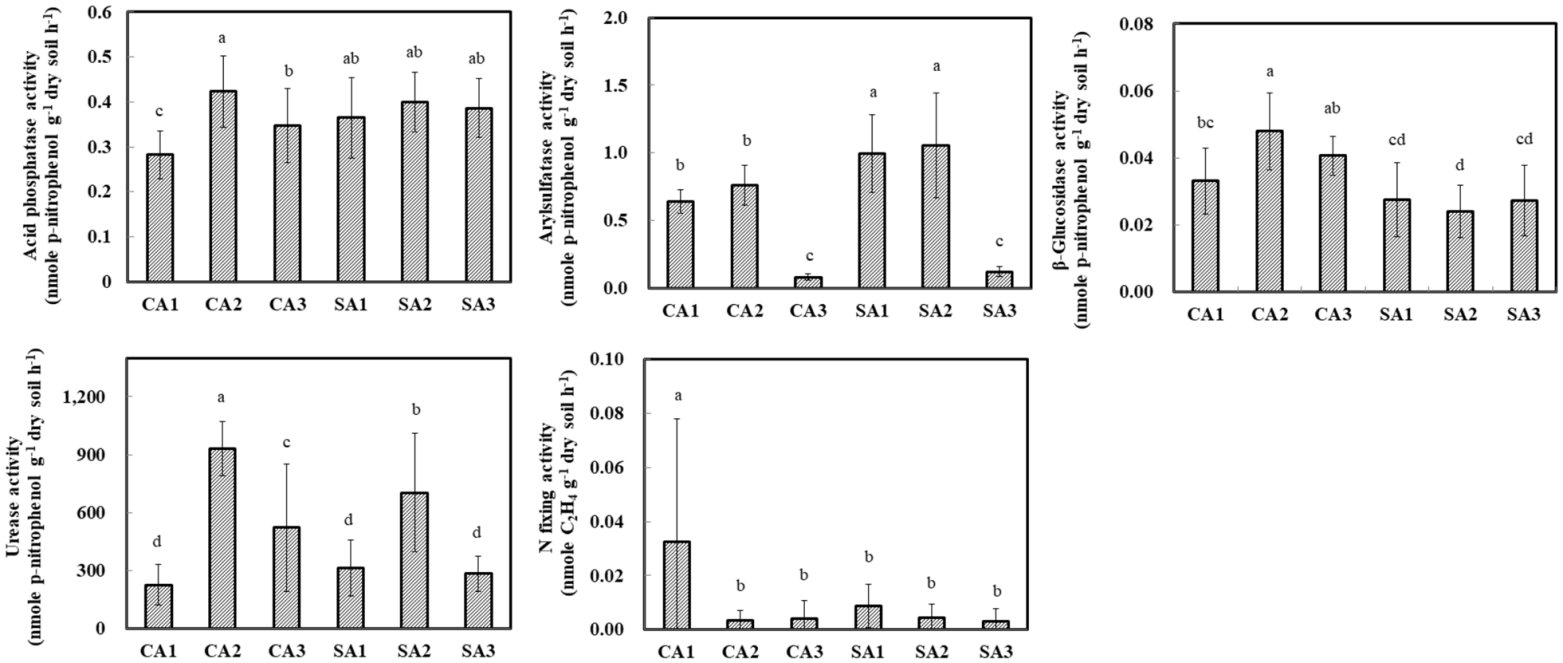
Temporal variations in the soil enzymatic activities of litchi orchards that were maintained under conventional (CA) and sustainable (SA) agricultural practices. 1, 2 and 3 indicate successive trimester samplings carried out on October 2016, January 2017 and April 2017, respectively. Acid phosphatase, arylsulfatase, and β-glucosidase activities were analyzed by the *p*-nitrophenol method. The N_2_-fixing activity was detected by the acetylene reduction method using gas chromatography. Results are shown as mean ± standard deviation. The different letters labeled at columns are significantly different according to Duncan’s test (P < 0.05) by IBM SPSS Statistics v20

The soil enzymatic activities of acid phosphatase, arylsulfatase, β-glucosidase, urease, and N_2_-fixing activities involved in phosphorus, sulfur, carbon, and nitrogen cycles were measured (Fig. 2). The result revealed that the enzymatic activities were affected not only by agricultural management but also temporal changes. Acid phosphatase and arylsulfatase in the SA soil had higher activity than in the CA soils. The enzymatic activity of arylsulfatase dropped in April 2017. By contrast, β-glucosidase and urease activities in the CA soil were higher than in the SA soils. Urease activity tended to be high in January 2017. The N_2_-fixing activity in soils was performed by the acetylene reduction assay. The result indicated that the N_2_-fixing activity in the CA soil of October 2016 was higher than that of the other soils. No significant difference was observed in the CA and SA soils of January 2017 and April 2017.

RM-ANOVA was used to assess the effect of agricultural management on different enzymatic activity across temporal change (Table 2). The result indicated significant variations in enzymatic activity with sampling time. However, a significant interaction between temporal change and agricultural management only appeared in the arylsulfatase, β-glucosidase, and urease activities.

**Table 2:** Significance of p-value for repeated measures ANOVA on acid phosphatase, arylsulfatase, β-glucosidase, urease, and N_2_-fixing activity from the litchi orchard soils

Model term	Enzymatic activity									
	Acid phosphatase		Arylsulfatase		β-Glucosidase		Urease		N_2_-fixing	
	***F***	***P***	***F***	***P***	***F***	***P***	***F***	***P***	***F***	***P***
Test of within-subjects effects										
Time	7.785	0.001**	111.941	< 0.001**	7.130	0.004**	34.217	< 0.001**	7.596	0.009**
Time × Management	2.835	0.069	3.918	0.027*	18.992	< 0.001**	3.586	0.047*	3.774	0.060
Test of between-subjects effects										
Intercept	1725.628	< 0.001**	310.086	< 0.001**	324.388	< 0.001**	738.748	< 0.001**	20.710	< 0.001**
Management	3.433	0.077	10.950	0.003**	14.856	0.001**	12.037	0.002**	3.692	0.068

Significance is indicated by **p-value < 0.01, and *p-value < 0.05

### Soil bacterial community through 16S rRNA analysis using Illumina MiSeq

To analyze the diversity of the bacterial community with the soil samples, the high-throughput sequencing was used for the hypervariable V6 region of the 16S rRNA by Illumina MiSEq. Raw reads with adapter contamination, ambiguous base, and low complexity were trimmed and removed. The average clean paired-reads and data were 54,526 reads and 31.176 Mb in each sample, respectively (Additional file 5: Table S3). Venn diagram represented the common and unique OTUs of CA and SA soil samples (Additional file 2: Fig. S2). The number of unique OTUs in CA and SA soils was obviously low in January 2017. In addition, the unique OTUs in the SA soil were considerably higher than those of the CA soil in October 2016 and January 2017.

**Fig. 3:**
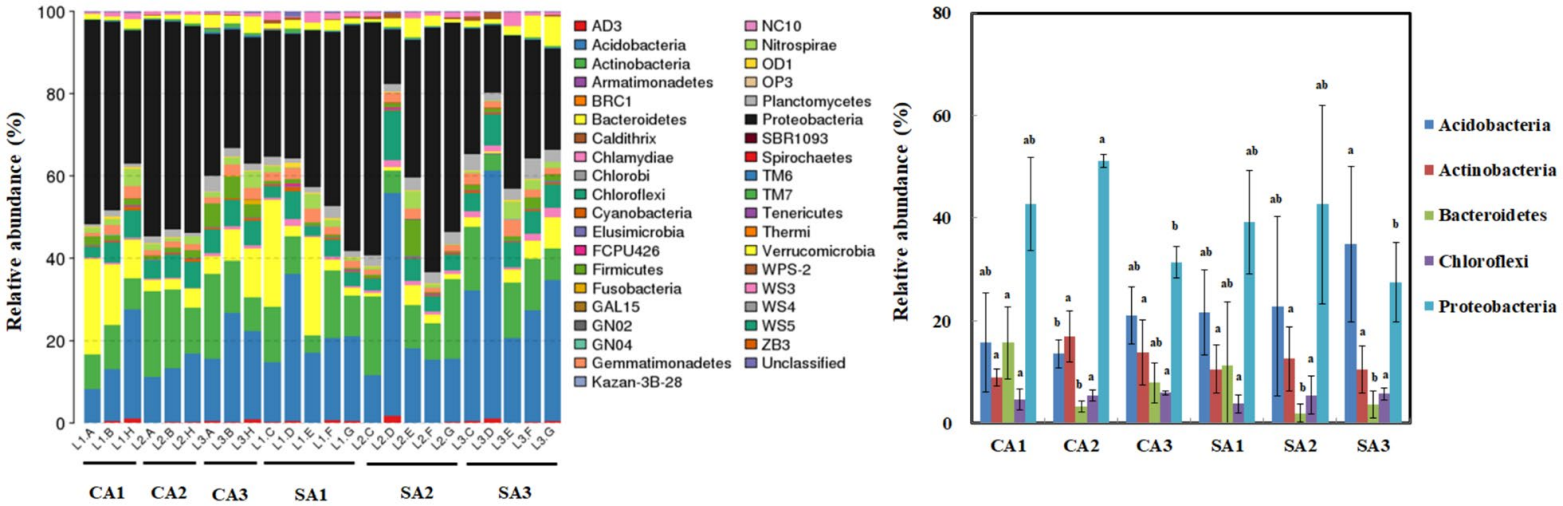
Temporal variation in the Phylum-level distribution of OTUs in the soil samples of litchi orchards that were maintained under conventional (CA) and sustainable (SA) agricultural practices. 1, 2 and 3 indicate successive trimester samplings carried out on October 2016, January 2017 and April 2017, respectively. Graph showing the taxonomic composition distribution was generated by the R software. The dominant phyla including Acidobacteria, Actinobacteria, Bacteroidetes, Chloroflexi, and Proteobacteria were further compared. Results are shown as mean ± standard deviation

The bacterial distribution in the soils was analyzed against the Greengenes database. The result revealed that Acidobacteria, Actinobacteria, Bacteroidetes, Chloroflexi, and Proteobacteria were the predominant phyla (Fig. 3). No significant difference was observed in the Actinobacteria and Chloroflexi between the CA and SA soils. The Acidobacteria in the SA soil was higher than that of CA soil. However, Bacteroidetes and Proteobacteria in the CA soil had a higher amount than that of SA soil. Moreover, the relative abundance of Acidobacteria, Bacteroidetes, and Proteobacteria was considerably influenced by sampling time. Relative abundance of Acidobacteria in the CA and SA soils was high in April 2017 than in October 2016 and January 2017, whereas the lower proportion of Proteobacteria was detected in April 2017. Additionally, the relative abundance of Bacteroidetes in October 2016 was 5.1- and 2.4-fold higher than that in January 2017 and April 2017, respectively. Further taxonomic division at the class-level indicated that Gammaproteobacteria was the most dominant with a relative abundance of 17.1% and 14.3% in the CA and SA soils on average, respectively.

**Fig. 4:**
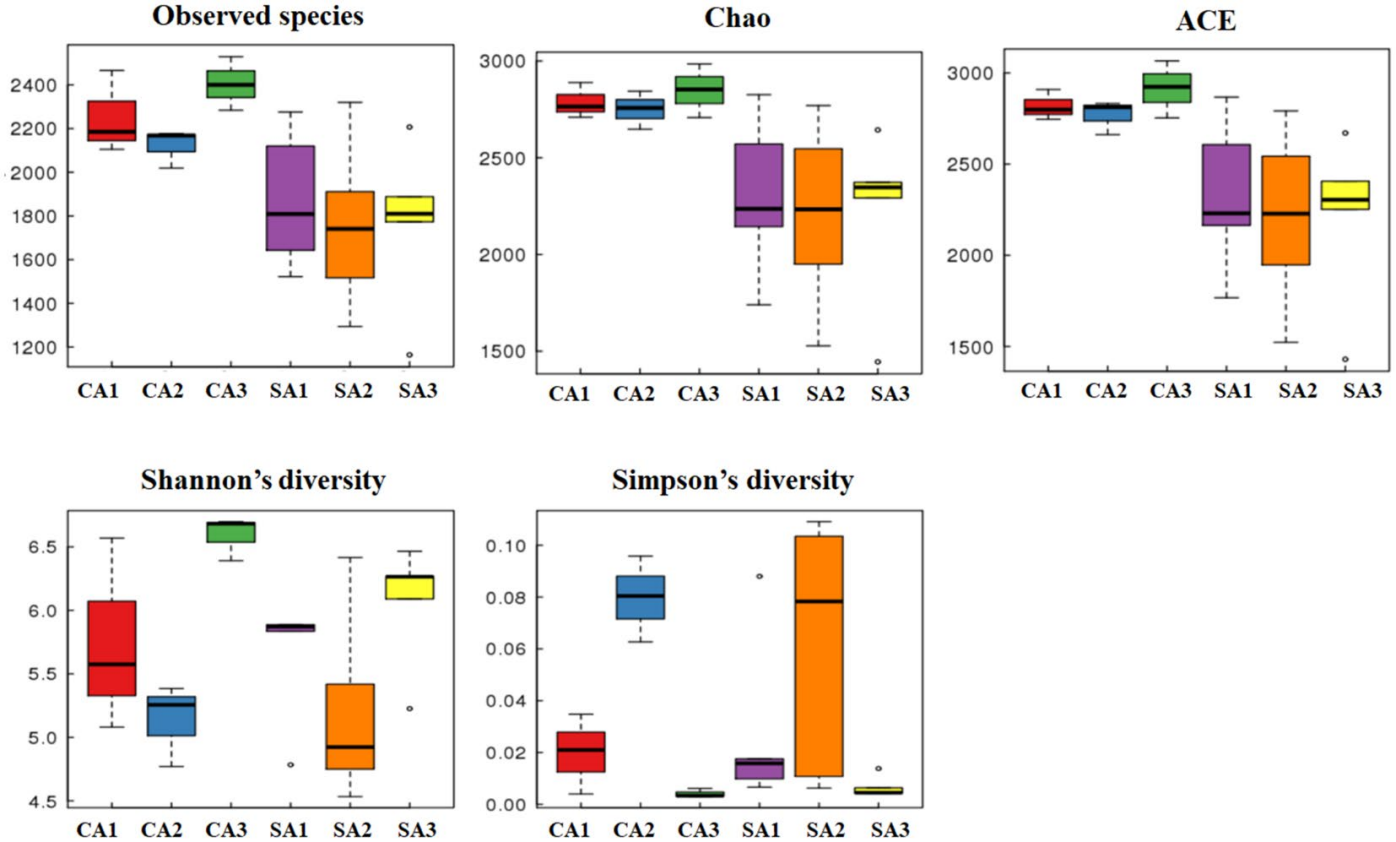
Temporal variations in the alpha diversity indices of soil bacteria inhabiting the litchi orchards that were maintained under conventional (CA) and sustainable (SA) agricultural practices. 1, 2 and 3 indicate successive trimester samplings carried out on October 2016, January 2017 and April 2017, respectively. The indices were calculated by Mothur (v1.31.2) and graphs were generated by the R software

**Fig. 5:**
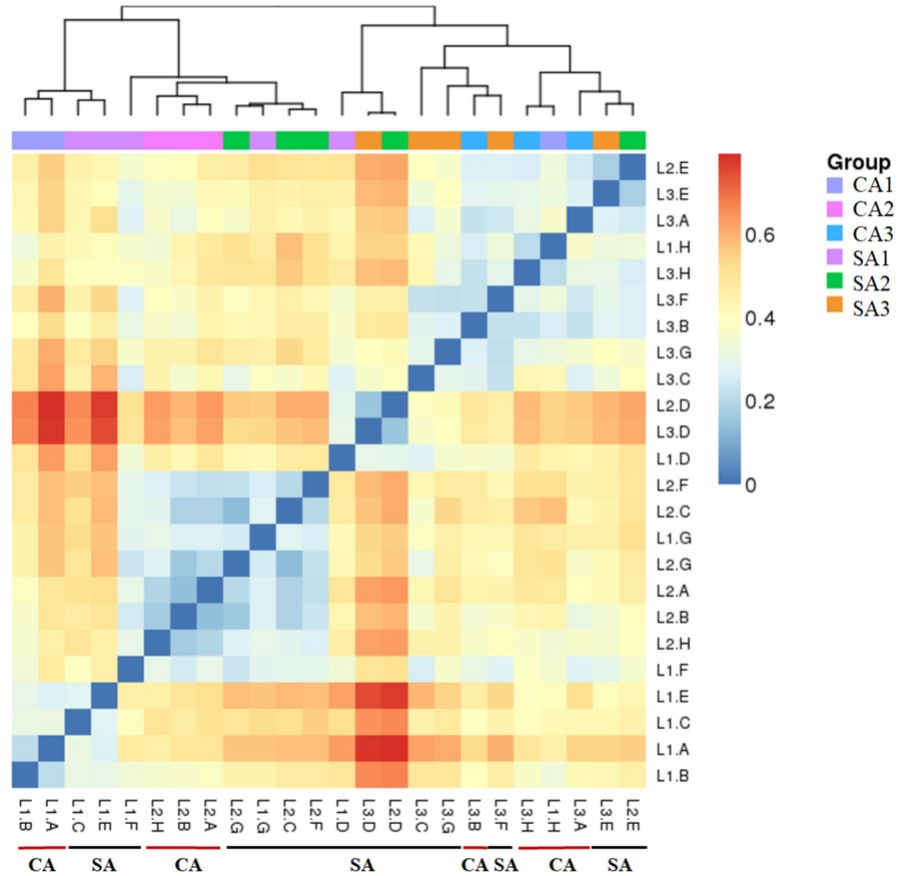
Temporal variations in the beta diversity of the soil bacteria inhabiting litchi orchards that were maintained under conventional (CA) and sustainable (SA) agricultural practices. 1, 2 and 3 indicate successive trimester samplings carried out on October 2016, January 2017 and April 2017, respectively. Beta diversity with weighted UniFrac analysis between October 2016 and April 2017 was measured by software QIIME (v1.80). Beta diversity heat map was generated by the R software in the NMF package

The observed species (SOBS) and alpha-diversity including Chao, ACE, Shannon, and Simpson were evaluated under the impact of agricultural management and temporal change (Fig. 4). SOBS, Chao, and ACE represent the species richness of community. The averages of SOBS, Chao, and ACE were 2259.22, 2795.26, and 2833.53 in the CA soil, and 1799.67, 2243.00, and 2248.71 in the SA soil. The result revealed that the CA soil had a higher amount of bacterial species and greater richness than that of SA soil. Nevertheless, the bacterial diversity was variable at different sampling time. The trend of bacterial diversity in both soils was following the order April 2017 > October 2016 > January 2017 according to the analysis of Shannon and Simpson indices. Beta-diversity with weighted UniFrac analysis was used to estimate the differences of soil samples in species complexity (Fig. 5). The result indicated that the five clusters can be divided, suggesting that the temporal changes distinguished from the soil samples in addition to the treatment of agricultural management.

Repeated measures analysis of variances (ANOVAs) was used to evaluate the effect of agricultural management on the bacterial predominant phyla and alpha diversity across temporal change (Additional file 6: Table S4). The result indicated that a significant difference with temporal change was observed in Acidobacteria and Proteobacteria. A marginally significant temporal change was apparent in Bacteroidetes. However, no significant agricultural management effect that differed over temporal change was perceived in these predominant phyla. On the other hand, no significant difference with time and agricultural management was found in the SOBS, Chao, and ACE index corresponding to bacterial richness (Additional file 7: Table S5). Nevertheless, a significant difference with time appeared in the Shannon and Simpson index corresponding to bacterial diversity.

### Relationship between the bacterial community, soil enzymatic activity, and soil properties

**Fig. 6:**
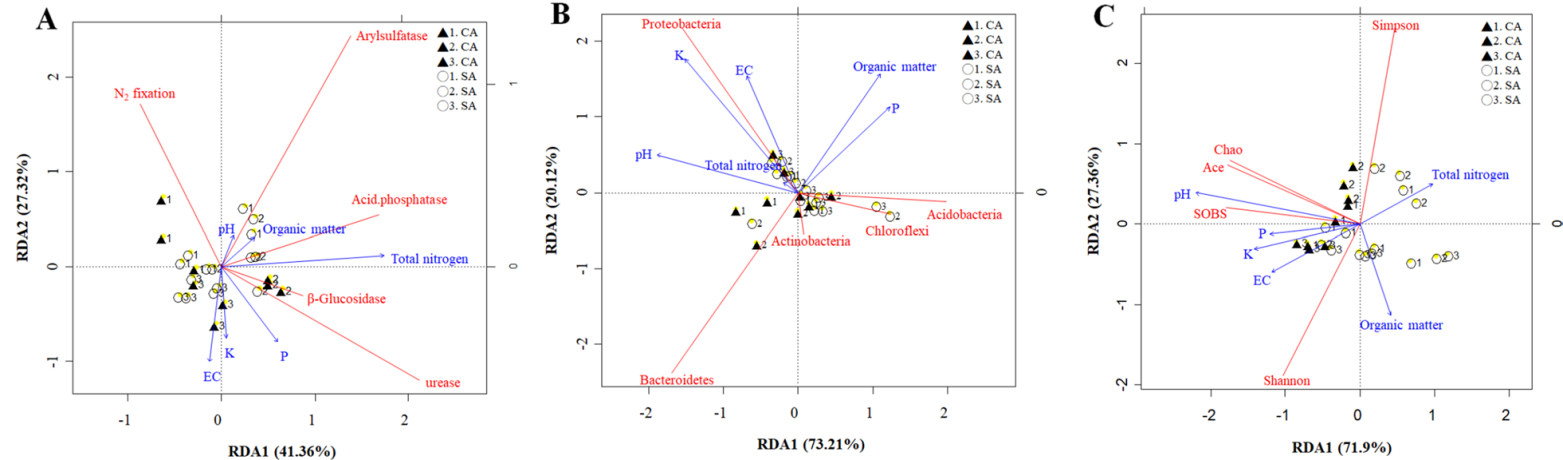
Redundancy analysis of the temporal variation in the soil characteristics of litchi orchards that were maintained under conventional (CA) and sustainable (SA) agricultural practices. 1, 2 and 3 indicate successive trimester samplings carried out on October 2016, January 2017 and April 2017, respectively. The comparison between the soil properties (pH, EC, K, total N, and OM), and **A** enzymatic activities, **B** the most abundant bacterial phyla, and **C** alpha diversity of OTUs was done by RDA. The RDA plots were generated by the R software in the vegan package

The relationship between bacterial community, enzymatic activity, and soil properties was further explored by redundancy analysis (Fig. 6). The RDA components interpreted 68.68%, 93.33%, and 99.26% of the variation in enzymatic activity, bacterial community, and diversity, respectively. Concerning the enzymatic activity, the first component explained 41.36% of the total variation (Fig. 6A). Soil samples were clustered along the sampling time. Acid phosphatase, β-glucosidase, and urease activities were related to total nitrogen in the soils of January 2017, whereas arylsulfatase activity occurred predominantly in the SA soil of October 2016 and January 2017 and negatively correlated with EC. Moreover, β-glucosidase was associated with the available phosphorus. Regarding the dominant phyla of bacteria, clustering between soil samples was not obvious (Fig. 6B). A related correlation was found in EC and available potassium with the abundance of Proteobacteria. However, the relationship of the other dominant bacteria, such as Acidobacteria, Actinobacteria, and Chloroflexi, and soil EC, and available potassium was the opposite. A similar result of the negative association was also observed between Bacteroidetes, and soil organic matter and available phosphorus. SOBS, Chao, and ACE indices were predominated in the CA soils and positively associated with soil pH (Fig. 6C). In addition, a positive correlation between bacteria diversity such as Shannon index, and soil EC and organic matter was dominantly perceived in the soils of October 2016 and April 2017.

Multivariate linear regression ANOVA with stepwise method was used to model the relationship between the enzymatic activity, bacterial community and soil properties. Pearson correlation was developed and shown (Table 3 and Additional file 8: Table S6). No significant association with soil property was observed in the N_2_-fixing activity and the dominant bacteria including Acidobacteria, Actinobacteria, Bacteroidetes, and Chloroflexi. In addition, under p-value < 0.05, acid phosphatase, arylsulfatase, β-glucosidase, SOBS, Chao, ACE, Shannon, and Simpson were used as the dependent variable in the multivariate linear regression with stepwise method. Equations were shown in Additional file 9: Table S7. On the other hand, the soil enzymatic activities were used as the dependent variable, and the dominant bacterial phyla were used as the independent variable in the stepwise multiple regression model. Under p-value < 0.05, only N_2_-fixing was a significant difference with the dominant bacteria (Additional file 9: Table S7).

**Table 3:** Pearson correlation between enzymatic activity and bacterial community

	Acidobacteria	Actinobacteria	Bacteroidetes	Chloroflexi	Proteobacteria
Acid phosphatase	0.168	0.049	–0.135	0.200	– 0.184
Arylsulfatase	– 0.286	0.103	0.106	– 0.059	0.369*
β-Glucosidase	– 0.147	–0.181	0.053	0.001	0.142
Urease	0.048	– 0.382*	0.263	– 0.079	–0.075
N-fixing	–0.311	– 0.135	0.425*	– 0.135	0.300

Significance is indicated by *p-value < 0.05

## Discussion

Temporal variation including climate conditions and soil properties is relatively common in different ecosystems with seasonal changes (Buscardo et al. 2018; Shigyo et al. 2019). Several kinds of research indicated that the observed species abundance and diversity of soil bacteria revealed the various patterns in the seasonal dynamics (Armstrong et al. 2016; Kivlin and Hawkes 2016; Murphy et al. 2016; Park et al. 2015; Shigyo et al. 2019). However, little attention has been done regarding the relationship between the soil bacterial community and enzymatic activity with the temporal changes under different agricultural practices. In this study, the soil bacterial community and enzymatic activity mediated by conventional and sustainable management in the litchi orchards were compared and determined. The litchi orchard in the SA soil has been planted for over 70 years without the application of pesticide, organic fertilizer, and chemical fertilizer. Because there has been no man-made interference for a long time, the SA soil was similar to the forest soil with a low pH through biological acidification or air pollution (Mao et al. 2017; Tamm and Hallbäcken 1986). The EC value fluctuated with fertilization in the CA soil whereas it was stable in the SA soil (Fig. 1). Moreover, the SA soil with old-growth litchi was typically rich in nitrogen, but poor in extractable phosphorus through soil leaching and immobilizing process (Vitousek et al. 2010). Although no fertilizer was applied in the SA soil, organic matter in the SA soil was equal to or slightly higher than that in the CA soil. This may result from a lot of litter and fruit decomposition residue. These soil properties indicated that long-term litchi cultivation resulted in a lowering of pH and reducing the available elements such as phosphorus in the soils. Similarly, the positive correlation between pH and available phosphorus was observed in the forest soil (Yang et al. 2018). Forest trees had a high influence on soil than that of perennial vegetation because of their longevity without soil management.

In the present study, different enzymatic activities revealed the various patterns depending on the sampling times and agricultural managements (Fig. 2). The patterns of RDA analysis did not synchronize between the enzymatic activities and bacterial community. The cluster among enzymatic activities, soil properties, and agricultural management practices was clearly distinguished (Fig. 6). According to the repeated measures ANOVA analysis, acid phosphatase, arylsulfatase, β-glucosidase, urease, and N_2_-fixing activity were all significantly affected by the temporal change (Table 2). Nevertheless, only arylsulfatase, β-glucosidase, and urease were significantly responded in the interaction of time and treatment. Although β-glucosidase is known to participate in the carbon cycle and associated with organic carbon (Dai et al. 2018; Xian et al. 2015), soil pH and phosphorus content were the dominant factors involved in the β-glucosidase activity in this study. According to the stepwise multiple regression model, the phosphorus explained 39% of the variation in the β-glucosidase activity (Additional file 9: Table S7, Eq. 1). Fatemi et al. indicated that the soil β-glucosidase activity was elevated in response to acute nutrient additions with phosphorus amendment, suggesting that inorganic phosphorus was important in regulating the soil β-glucosidase in the acid forest soils (Fatemi et al. 2016). Besides, phosphorus was deficient in acidic soil and complexed with iron and aluminum (Chen et al. 2006). Therefore, the soil β-glucosidase was positively associated with soil pH and phosphorus in the litchi orchards. The acid phosphatase and urease activities involved in mineralization of organic phosphorus and urea hydrolysis were related to the total nitrogen in the CA and SA soils of January 2017 by the RDA analysis. Under the probability of F < 0.05, the multiple regression model picked out total nitrogen as a significant predictor of acid phosphatase for describing 29.5% of the variation (Additional file 9: Table S7, Eq. 2). This result was consistent with the different soil environments such as the coastal saline soil, indicating the close relationship between soil nitrogen content and enzymatic activity (Xie et al. 2017). In addition, the increase of diazotrophs abundance was probably associated with raising the activity of acid phosphatase and urease, requiring a considerable amount of nitrogen (Lemanowicz 2011; Xie et al. 2017). However, N_2_-fixing activity across temporal change was not coincident with the activity of acid phosphatase and urease in this study. This finding was similar to some studies which have shown that high nitrogen levels did not affect nitrogen fixation activity and the abundance of diazotrophic communities instead (Barron et al. 2008; Matson et al. 2015). Hence, the association of these enzymatic activities is worthy of further investigation.

Arylsulfatase can catalyze the hydrolysis of various substrates attached to the free sulfate group (Pettit et al. 1977). A positive relationship between the total nitrogen and arylsulfatase activity was observed in the SA soils (Additional file 9: Table S7, Eq. 3). However, the arylsulfatase activity was significantly dropped in the CA and SA soils of April 2017. Also, the atmospheric relative humidity and Proteobacteria relative abundance at sampling time were respectively declined in April 2017. This may be due to climatic effect on soil microorganisms (Mandal and Neenu 2012). It was generally believed that the climate change of temperature contributing to global warming was an important determinant for the regulation of soil biological structure and function. Nevertheless, the relative humidity related to water vapor can directly influence infrared radiation and indirectly change atmospheric temperature (Tullus et al. 2012). In this study, the relationship among the relative humidity, temperature, bacterial community and enzymatic activity could be explored by the Pearson correlation (Additional file 10: Table S8 and Additional file 11: Table S9). The result indicated that the temperature had a significantly positive correlation with the Shannon and Simpson index. By contrast, the relative humidity had a significantly negative correlation with the Shannon and Simpson index. On the other hand, the temperature had a significantly negative correlation with the acid phosphatase, arylsulfatase and urease. Nevertheless, the relative humidity had a significantly positive correlation with the arylsulfatase. Thus, the relative humidity and temperature could alter soil bacterial community and enzymatic activity.

Concerning the bacterial community, the dominant phyla were spatially stable but varied depending on temporal change such as Acidobacteria and Proteobacteria by the repeated measures ANOVA analysis (Additional file 6: Table S4). Although the in-depth analysis of orchard soil in Taiwan was relatively rare, the dominant phyla of litchi soil was consistent with montane forest ecosystem in Taiwan (Lin et al. 2010). In addition, no significant difference under different agricultural management across time was observed in the bacterial richness such as the SOBS, Chao, and ACE index (Additional file 7: Table S5). However, the bacterial diversity, Shannon and Simpson index, was significantly influenced by temporal change but not by agricultural management. Our results were significantly different from the analysis of neotropical rainfall and desert soil (Armstrong et al. 2016; Kivlin and Hawkes 2016). In the neotropical rainfall soil, a significant interaction between temporal change and vegetation type was perceived in bacterial richness and diversity (Kivlin and Hawkes 2016). By contrast, bacterial richness was spatially more variable than that of temporal variability in the desert soil (Armstrong et al. 2016). This suggested that the different environmental soil had different consequences on the bacterial structure. Additionally, the bacterial diversity, calculated by the Shannon and Simpson index, was lower in January 2017 than the other sampling times. January 2017 was the lowest temperature recorded in all sampling times. This suggested that the seasonal effect with temperature change obviously affected the soil bacterial diversity. However, our result was contrary to the results of the previous studies, indicating low bacterial richness and diversity in the summer (Jung et al. 2012; Rasche et al. 2011).

Taking together our results imply that the enzymatic activity was more sensitive to temporal change and agricultural management than that of bacterial richness and diversity. Several environmental factors such as climate change, soil organic matter, pH, and water-soluble ions can examine the temporal change of the microbial community (Armstrong et al. 2016; Kivlin and Hawkes 2016; Shigyo et al. 2019). According to the Pearson correlation and RDA between bacterial richness and soil properties, soil pH and extractable potassium were significantly affect the SOBS, ACE, and Chao index in the present study (Additional file 8: Table S6) (Fig. 6). Soil pH was considered to be the main factor that impacting the bacterial community (Lammel et al. 2018; Lauber et al. 2009; Shen et al. 2019). This phenomenon was often found in the acidity forest soil due to the leaching, organic matter decay and acidic parent material. Moreover, potassium is the primary prerequisite for an organism’s growth because it is involved in a regulator of cytoplasmic pH and cell turgor (Booth 1985). A similar result was also observed in the Shigyo et al. study, indicating that the shape of the microbial community was influenced by water-soluble potassium and phosphorus (Shigyo et al. 2019). Further modeling the soil bacterial community and property by multivariate linear regression ANOVA with the stepwise method can find the possible correlation and variance. No significant relationship was perceived in the dominant bacteria such as Acidobacteria, Actinobacteria, Bacteroidetes, Chloroflexi, and Proteobacteria under the probability of F < 0.05. Nevertheless, among the six quantified soil properties, the multiple regression model picked out pH and EC as the significant predictor of SOBS, Chao, ACE, and Shannon. Because the old-growth litchi in the SA has resulted in biological acidification with a low pH of the soil and was similar to the forest soil, it was an inverse effect on plants with the deficiency of nutrients such as phosphorus (Mao et al. 2017; Tamm and Hallbäcken 1986). In this study, the soil pH and EC as the significant predictors explained 76.9% and 33.8% of the variation in the SOBS and Shannon index, respectively (Additional file 9: Table S7, Eqs. 4–5). In addition to soil pH and EC, it also contained phosphorus content, which can explain 77.1% and 79% of the variation in the Chao and ACE index (Additional file 9: Table S7, Eqs. 6–7). On the other hand, the Simpson index represents the probability that two randomly selected individuals belong to different species, indicating the bigger the value, the lower diversity (McCune and Grace 2002). The multiple regression model picked out organic matter and total nitrogen as the significant predictors of Simpson instead (Additional file 9: Table S7, Eq. 8). Because no ideal diversity index was suggested (Morris et al. 2014), SOBS, Chao, ACE, and Shannon may appear interchangeable by the multiple regression model and were preferable. Furthermore, a significant association was observed between soil enzymatic activity and dominant phyla. The multiple regression model picked out N_2_-fixing activity as a significant predictor of Bacteroidetes for explaining 18% of the variation. In the Bacteroidetes, the N_2_-fixing bacteria revealed the presence of *Flavobacterium* in this study, which may be contributed by a nitrogen-fixing bacterium, *Flavobacterium nitrogenifigens* (Kampfer et al. 2015). This result of cross-over analysis could explore the association of soil enzymatic activity with bacterial composition in the soil.

## Conclusions

A long-term sustainable agriculture in litchi orchards was not necessarily beneficial to soil health, resulting in low pH and phosphorus of soil. However, the application of chemical fertilizer will accelerate soil acidification and cause serious damage to the soil. Our results indicated that the soil enzymatic activity including acid phosphatase, arylsulfatase, β-glucosidase, urease, and nitrogen fixation was significantly influenced by the temporal change in the litchi orchards. Also, the significant effect of agricultural management across time on the arylsulfatase, β-glucosidase, and urease was observed. Regarding the bacterial community, no interaction of agricultural management and temporal changes on bacterial alpha diversity and dominant phyla was found, but bacterial diversity, Shannon and Simpson, and dominant phyla, Acidobacteria and Proteobacteria, were significantly influenced by time. These results suggested that soil enzyme activity was more susceptible to temporal change and agricultural management than that of bacterial richness and diversity. In addition, soil enzymatic activity was widely affected by soil environmental factors due to the dynamics and turn-over times of enzymes variously involved in enzyme-mediated decay with their function and origin. By contrast, soil pH directly affecting extractable elements could fluctuate the bacterial richness and diversity. Nevertheless, it was difficult to interpret the correlation of dominant phyla with the soil property, which only a significant relationship between Proteobacteria and potassium was perceived. Thus, the temporal change involved in soil property could result in different degrees of impact on soil enzymes and bacteria.

## Supplementary Information

**Additional file 1: Figure S1.** The map of the litchi orchards including CA and SA managements.

**Additional file 2: Figure S2.** The Venn chart of OTUs obtained from CA and SA soils of litchi orchards between October 2016 and April 2017.

**Additional file 3: Table S1.** Agricultural management of litchi orchards by conventional (CA) and sustainable agriculture (SA).

**Additional file 4: Table S2.** Pearson correlation analysis of soil properties.

**Additional file 5: Table S3.** 16S rRNA reads summary of soil DNA obtained from CA and SA soils of litchi orchards between October 2016 and April 2017 according to the Illumina MiSeq analysis.

**Additional file 6: Table S4.** Significance of p-value for repeated measures ANOVA on bacterial distribution including Acidobacteria, Actinobacteria, Bacteroidetes, Chloroflexi, and Proteobacteria.

**Additional file 7: Table S5.** Significance of p-value for repeated measures ANOVA on alpha diversity of bacteria including SOBS, Chao, ACE, Shannon, and Simpson indices.

**Additional file 8: Table S6.** Pearson correlation between enzymatic activity, bacterial community, and soil properties.

**Additional file 9: Table S7.** Modeling the enzymatic activity and bacterial community with soil chemical properties and dominant bacteria by multivariate linear regression ANOVA with the stepwise method.

**Additional file 10: Table S8.** Pearson correlation between temperature, relative humidity, and bacterial community.

**Additional file 11: Table S9.** Pearson correlation between temperature, relative humidity, and enzymatic activity.

## Data Availability

The data used and analyzed in this study can be provided from the corresponding author for scientific, non-profit purpose.
