# Supplementary material for: Agricultural management and cultivation period alter soil enzymatic activity and bacterial diversity in litchi (Litchi chinensis Sonn.) orchards
Source: Bot Stud. 2021 Sep 26;62:13. doi: 10.1186/s40529-021-00322-9 (PMC8473471; doi:10.1186/s40529-021-00322-9)
Supplement: Supplementary file 1 — Additional file 1: Figure S1. The map of the litchi orchards including CA and SA managements. [file 40529_2021_322_MOESM1_ESM.docx]

**Figure S1.** The map of the litchi orchards including CA and SA managements. The litchi orchards were located at 23°29'5.1288''N, 120°28'12.2592''E. The red frames indicate the experimental fields. A, B, and H refer to the CA management. C, D, E, F, and G refer to the SA management. The map was obtained from the Google map.
