# Supplementary material for: Agricultural management and cultivation period alter soil enzymatic activity and bacterial diversity in litchi (Litchi chinensis Sonn.) orchards
Source: Bot Stud. 2021 Sep 26;62:13. doi: 10.1186/s40529-021-00322-9 (PMC8473471; doi:10.1186/s40529-021-00322-9)
Supplement: Supplementary file 2 — Additional file 2: Figure S2. The Venn chart of OTUs obtained from CA and SA soils of litchi orchards between October 2016 and April 2017. [file 40529_2021_322_MOESM2_ESM.docx]

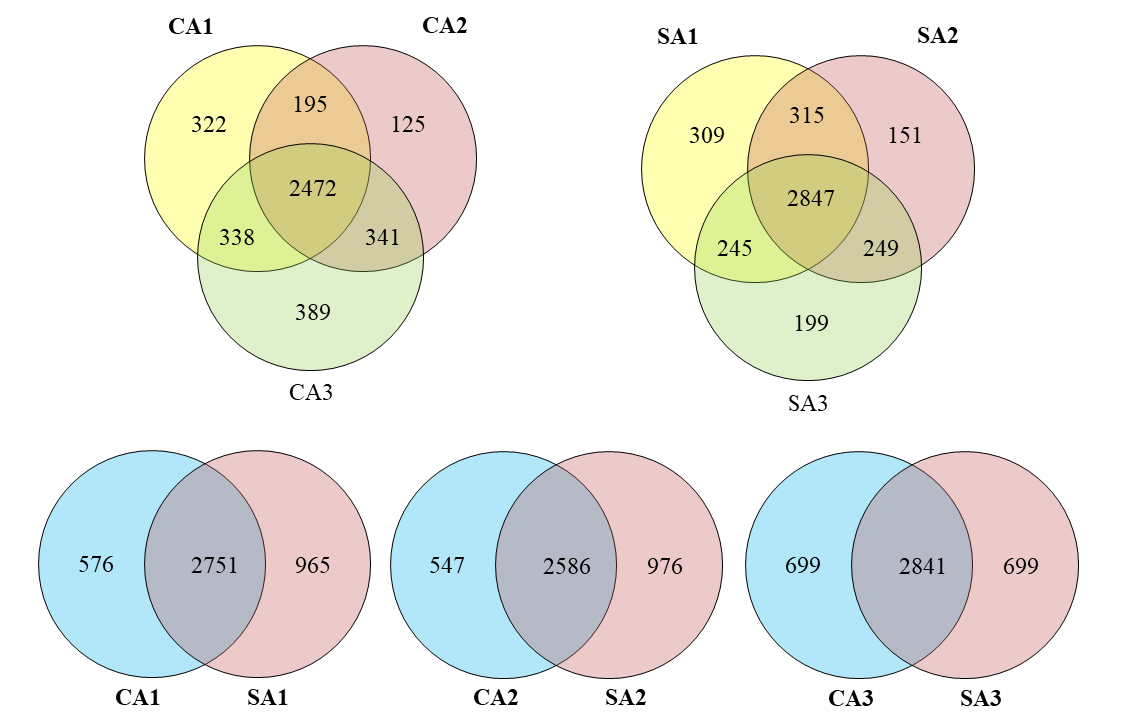


**Figure S2.** The Venn chart of OTUs obtained from CA and SA soils of litchi orchards between October 2016 and April 2017. 1, 2 and 3 indicate successive trimester samplings carried out on October 2016, January 2017 and April 2017, respectively.
