# Supplementary material for: Agricultural management and cultivation period alter soil enzymatic activity and bacterial diversity in litchi (Litchi chinensis Sonn.) orchards
Source: Bot Stud. 2021 Sep 26;62:13. doi: 10.1186/s40529-021-00322-9 (PMC8473471; doi:10.1186/s40529-021-00322-9)
Supplement: Supplementary file 3 — Additional file 3: Table S1. Agricultural management of litchi orchards by conventional (CA) and sustainable agriculture (SA). [file 40529_2021_322_MOESM3_ESM.docx]

**Table S1.** Agricultural management of litchi orchards by conventional (CA) and sustainable agriculture (SA).

| CA | |
| --- | --- |
| Chemical fertilizer | 1. Heading stage: N:P_2_O_5_:K_2_O:MgO = 15:15:15:4; 1.5 kg per plant. 2. Flower bud differentiation: Potassium dihydrogen phosphate, 1 g/1000 mL with four times. 3. Seven weeks after blossoming: N:P_2_O_5_:K_2_O:MgO = 15:15:15:4; 0.6-0.9 kg per plant. 4. After harvest: N:P_2_O_5_:K_2_O:MgO = 15:15:15:4; 1.5 kg per plant. |
| Organic fertilizer | N:P_2_O_5_:K_2_O:organic matter = 1.5:0.9:1.5:55; 10 kg per plant. |
| Pesticide | 1. Heading stage: 85% Carbaryl, 1 g/850 mL once; 2.4% Decamethrin, 1 g/ 1500 mL once. 2. Fruiting stage: 85% Carbaryl, 1 g/850 mL; 2.4% Decamethrin, 1 g/ 1500 mL once; 72% Curzate-M, 1 g/500 mL with five times. |
| Orchard management | Weeding with herbicide; Pruning after harvest; Trimming the height of the plant to 1.8 m; Ring girdling in November. |
| SA | |
| Chemical fertilizer | None |
| Organic fertilizer | None |
| Pesticide | None |
| Orchard management | Treating herbicide in the drought; No pruning except dead branches; Fruit was not harvested and dropped off naturally. |
