# Supplementary material for: Agricultural management and cultivation period alter soil enzymatic activity and bacterial diversity in litchi (Litchi chinensis Sonn.) orchards
Source: Bot Stud. 2021 Sep 26;62:13. doi: 10.1186/s40529-021-00322-9 (PMC8473471; doi:10.1186/s40529-021-00322-9)
Supplement: Supplementary file 4 — Additional file 4: Table S2. Pearson correlation analysis of soil properties. [file 40529_2021_322_MOESM4_ESM.docx]

**Table S2.** Pearson correlation analysis of soil properties. Significance is indicated by **p-value < 0.01, and *p-value < 0.05. O.M. and T.N. are organic matter and total nitrogen, respectively.

| **pH** | –0.220 | –0.085 | –0.099 | **0.549**** | 0.165 | **0.753**** | **0.660**** | –**0.368*** | **0.388*** | **0.705**** | **0.505*** |
| --- | --- | --- | --- | --- | --- | --- | --- | --- | --- | --- | --- |
|  | **EC** | –0.005 | –0.189 | 0.140 | **0.714**** | 0.204 | –0.181 | –0.185 | –0.204 | –0.019 | 0.312 |
|  |  | **O.M.** | **0.510*** | 0.173 | 0.093 | 0.245 | –0.020 | –0.092 | –0.081 | 0.165 | 0.049 |
|  |  |  | **T.N.** | 0.228 | –0.009 | 0.138 | –0.076 | 0.233 | –**0.449*** | 0.181 | 0.199 |
|  |  |  |  | **P** | 0.230 | **0.548**** | 0.241 | –**0.570**** | 0.106 | **0.923**** | **0.731**** |
|  |  |  |  |  | **K** | **0.477**** | –0.063 | –0.286 | –0.156 | 0.153 | 0.225 |
|  |  |  |  |  |  | **Ca** | **0.728**** | –0.336 | 0.298 | **0.637**** | **0.639**** |
|  |  |  |  |  |  |  | **Mg** | –0.110 | **0.606**** | **0.465*** | **0.463*** |
|  |  |  |  |  |  |  |  | **Fe** | –**0.440*** | –**0.557**** | –0.287 |
|  |  |  |  |  |  |  |  |  | **Mn** | 0.291 | 0.126 |
|  |  |  |  |  |  |  |  |  |  | **Cu** | **0.680**** |
|  |  |  |  |  |  |  |  |  |  |  | **Zn** |
