# Supplementary material for: Agricultural management and cultivation period alter soil enzymatic activity and bacterial diversity in litchi (Litchi chinensis Sonn.) orchards
Source: Bot Stud. 2021 Sep 26;62:13. doi: 10.1186/s40529-021-00322-9 (PMC8473471; doi:10.1186/s40529-021-00322-9)
Supplement: Supplementary file 5 — Additional file 5: Table S3. 16S rRNA reads summary of soil DNA obtained from CA and SA soils of litchi orchards between October 2016 and April 2017 according to the Illumina MiSeq analysis. [file 40529_2021_322_MOESM5_ESM.docx]

**Table S3.** 16S rRNA reads summary of soil DNA obtained from CA and SA soils of litchi orchards between October 2016 and April 2017 according to the Illumina MiSeq analysis.

| Sampling time | Samples | Reads length (bp) | Raw data (Mb) | Clean data (Mb) | Raw reads | Clean reads | OTU number |
| --- | --- | --- | --- | --- | --- | --- | --- |
| 2016/10 | CA-A | 294:296 | 34.20 | 31.01 | 57970×2 | 54383×2 | 2105 |
|  | CA-B | 296:296 | 37.38 | 31.21 | 63145×2 | 54961×2 | 2185 |
|  | CA-H | 294:297 | 34.81 | 31.41 | 58901×2 | 55135×2 | 2466 |
|  | SA-C | 297:296 | 35.22 | 31.20 | 59385×2 | 54521×2 | 2276 |
|  | SA-D | 298:296 | 35.14 | 31.28 | 59152×2 | 54417×2 | 1522 |
|  | SA-E | 299:296 | 34.99 | 31.45 | 58812×2 | 54768×2 | 2120 |
|  | SA-F | 300:297 | 34.89 | 31.44 | 58450×2 | 54540×2 | 1809 |
|  | SA-G | 293:297 | 35.41 | 31.11 | 60021×2 | 54793×2 | 1643 |
| 2017/01 | CA-A | 296:297 | 38.71 | 31.05 | 65272×2 | 54791×2 | 2019 |
|  | CA-B | 297:297 | 36.15 | 31.24 | 60863×2 | 54885×2 | 2177 |
|  | CA-H | 294:298 | 35.01 | 30.77 | 59136×2 | 54167×2 | 2168 |
|  | SA-C | 298:297 | 36.03 | 31.37 | 50547×2 | 54827×2 | 1911 |
|  | SA-D | 299:297 | 35.31 | 31.32 | 59238×2 | 54606×2 | 1294 |
|  | SA-E | 300:300 | 34.30 | 30.77 | 57167×2 | 53130×2 | 2320 |
|  | SA-F | 300:298 | 35.70 | 31.39 | 59699×2 | 54652×2 | 1741 |
|  | SA-G | 293:298 | 37.60 | 31.10 | 63622×2 | 54948×2 | 1517 |
| 2017/04 | CA-A | 293:300 | 34.81 | 30.85 | 58707×2 | 53984×2 | 2284 |
|  | CA-B | 297:298 | 36.51 | 31.42 | 61362×2 | 55005×2 | 2529 |
|  | CA-H | 294:299 | 34.59 | 30.93 | 58331×2 | 54190×2 | 2400 |
|  | SA-C | 298:298 | 35.84 | 30.97 | 60136×2 | 53856×2 | 1773 |
|  | SA-D | 299:298 | 36.78 | 31.49 | 61607×2 | 54989×2 | 1164 |
|  | SA-E | 300:298 | 36.85 | 31.36 | 61626×2 | 54648×2 | 2207 |
|  | SA-F | 300:299 | 34.80 | 31.25 | 58092×2 | 54221×2 | 1888 |
|  | SA-G | 293:299 | 37.12 | 30.77 | 62695×2 | 54217×2 | 1810 |
