# Supplementary material for: Agricultural management and cultivation period alter soil enzymatic activity and bacterial diversity in litchi (Litchi chinensis Sonn.) orchards
Source: Bot Stud. 2021 Sep 26;62:13. doi: 10.1186/s40529-021-00322-9 (PMC8473471; doi:10.1186/s40529-021-00322-9)
Supplement: Supplementary file 6 — Additional file 6: Table S4. Significance of p-value for repeated measures ANOVA on bacterial distribution including Acidobacteria, Actinobacteria, Bacteroidetes, Chloroflexi, and Proteobacteria. [file 40529_2021_322_MOESM6_ESM.docx]

**Table S4.** Significance of p-value for repeated measures ANOVA on bacterial distribution including Acidobacteria, Actinobacteria, Bacteroidetes, Chloroflexi, and Proteobacteria.

| Model term | Bacterial distribution | | | | | | | | | |
| --- | --- | --- | --- | --- | --- | --- | --- | --- | --- | --- |
|  | Acidobacteria | | Actinobacteria | | Bacteroidetes | | Chloroflexi | | Proteobacteria | |
|  | ***F*** | ***P*** | ***F*** | ***P*** | ***F*** | ***P*** | ***F*** | ***P*** | ***F*** | ***P*** |
| Test of within-subjects effects |  |  |  |  |  |  |  |  |  |  |
| Time | 6.331 | **0.013*** | 2.743 | 0.104 | 5.084 | 0.061 | 2.498 | 0.124 | 6.382 | **0.013*** |
| Time × Management | 1.025 | 0.388 | 1.072 | 0.373 | 0.113 | 0.762 | 0.891 | 0.436 | 0.171 | 0.845 |
| Test of between-subjects effects |  |  |  |  |  |  |  |  |  |  |
| Intercept | 27.494 | **0.002**** | 82.485 | **<0.001**** | 35.065 | **0.001**** | 55.087 | **<0.001**** | 161.974 | **<0.001**** |
| Management | 1.365 | 0.287 | 0.712 | 0.431 | 1.724 | 0.237 | 0.036 | 0.855 | 0.776 | 0.412 |

Significance is indicated by **p-value < 0.01, and *p-value < 0.05. *F* and *P* indicates the probability and significance test.
