# Supplementary material for: Agricultural management and cultivation period alter soil enzymatic activity and bacterial diversity in litchi (Litchi chinensis Sonn.) orchards
Source: Bot Stud. 2021 Sep 26;62:13. doi: 10.1186/s40529-021-00322-9 (PMC8473471; doi:10.1186/s40529-021-00322-9)
Supplement: Supplementary file 7 — Additional file 7: Table S5. Significance of p-value for repeated measures ANOVA on alpha diversity of bacteria including SOBS, Chao, ACE, Shannon, and Simpson indices. [file 40529_2021_322_MOESM7_ESM.docx]

**Table S5.** Significance of p-value for repeated measures ANOVA on alpha diversity of bacteria including SOBS, Chao, ACE, Shannon, and Simpson indices.

| Model term | Alpha diversity | | | | | | | | | | | | |
| --- | --- | --- | --- | --- | --- | --- | --- | --- | --- | --- | --- | --- | --- |
|  | SOBS | | Chao | | | ACE | | | | Shannon | | Simpson | |
|  | ***F*** | ***P*** | | ***F*** | ***P*** | | ***F*** | ***P*** | ***F*** | | ***P*** | ***F*** | ***P*** |
| Test of within-subjects effects |  |  | |  |  | |  |  |  | |  |  |  |
| Time | 2.024 | 0.175 | | 0.353 | 0.709 | | 0.529 | 0.602 | 9.585 | | **0.003**** | 10.641 | **0.002**** |
| Time × Management | 1.885 | 0.194 | | 0.349 | 0.712 | | 0.703 | 0.514 | 0.692 | | 0.520 | 0.435 | 0.657 |
| Test of between-subjects effects |  |  | |  |  | |  |  |  | |  |  |  |
| Intercept | 389.136 | **<0.001**** | | 386.770 | **<0.001**** | | 380.278 | **<0.001**** | 1828.267 | | **<0.001**** | 24.436 | **0.003**** |
| Management | 4.988 | 0.067 | | 4.647 | 0.075 | | 5.036 | 0.066 | 0.460 | | 0.523 | 0.038 | 0.851 |

Significance is indicated by **p-value < 0.01, and *p-value < 0.05. *F* and *P* indicates the probability and significance test.
