# Supplementary material for: Agricultural management and cultivation period alter soil enzymatic activity and bacterial diversity in litchi (Litchi chinensis Sonn.) orchards
Source: Bot Stud. 2021 Sep 26;62:13. doi: 10.1186/s40529-021-00322-9 (PMC8473471; doi:10.1186/s40529-021-00322-9)
Supplement: Supplementary file 8 — Additional file 8: Table S6. Pearson correlation between enzymatic activity, bacterial community, and soil properties. [file 40529_2021_322_MOESM8_ESM.docx]

**Table S6.** Pearson correlation between enzymatic activity, bacterial community, and soil properties.

|  | pH | EC | Organic matter | Total nitrogen | P | K |
| --- | --- | --- | --- | --- | --- | --- |
| Acid phosphatase | –0.021 | –0.172 | 0.278 | **0.543**** | 0.174 | –0.245 |
| Arylsulfatase | 0.116 | **–0.452*** | 0.163 | **0.452*** | –0.249 | –0.281 |
| β-Glucosidase | **0.607**** | 0.156 | 0.181 | 0.154 | **0.624**** | 0.266 |
| Urease | –0.111 | 0.184 | –0.132 | **0.555**** | 0.148 | 0.290 |
| N_2_-fixing | 0.221 | –0.187 | –0.066 | –0.276 | –0.305 | –0.055 |
| Acidobacteria | –0.260 | –0.124 | 0.161 | 0.082 | 0.147 | –0.151 |
| Actinobacteria | 0.024 | –0.109 | –0.051 | –0.125 | –0.081 | –0.108 |
| Bacteroidetes | 0.116 | –0.183 | –0.322 | 0.046 | –0.309 | –0.086 |
| Chloroflexi | –0.129 | –0.130 | 0.082 | –0.101 | 0.019 | –0.201 |
| Proteobacteria | 0.258 | 0.227 | 0.141 | 0.053 | 0.003 | **0.392*** |
| SOBS | **0.699**** | **0.362*** | –0.137 | –0.244 | **0.384*** | **0.456*** |
| Chao | **0.731**** | 0.257 | –0.299 | –0.258 | 0.332 | **0.362*** |
| ACE | **0.727**** | 0.281 | –0.284 | –0.255 | 0.330 | **0.387*** |
| Shannon | 0.268 | **0.444*** | 0.305 | –0.294 | **0.343*** | **0.388*** |
| Simpson | –0.013 | –0.305 | **–0.432*** | 0.335 | –0.069 | –0.210 |

Significance is indicated by **p-value < 0.01, and *p-value < 0.05.
