# Supplementary material for: Agricultural management and cultivation period alter soil enzymatic activity and bacterial diversity in litchi (Litchi chinensis Sonn.) orchards
Source: Bot Stud. 2021 Sep 26;62:13. doi: 10.1186/s40529-021-00322-9 (PMC8473471; doi:10.1186/s40529-021-00322-9)
Supplement: Supplementary file 9 — Additional file 9: Table S7. Modeling the enzymatic activity and bacterial community with soil chemical properties and dominant bacteria by multivariate linear regression ANOVA with the stepwise method. [file 40529_2021_322_MOESM9_ESM.docx]

**Table S7.** Modeling the enzymatic activity and bacterial community with soil chemical properties and dominant bacteria by multivariate linear regression ANOVA with the stepwise method.

| Equations | Model |  |
| --- | --- | --- |
| β-Glucosidase = 0.015 + 0.05 × P | r^2^ = 0.390, F = 14.041, p-value = 0.001 | (1) |
| Acid phosphatase = 0.212+0.106 × Total nitrogen | r^2^ = 0.295, F = 9.186, p-value = 0.006 | (2) |
| Arylsulfatase = 0.127 + 0.463 × Total nitrogen – 0.002 × EC | r^2^ = 0.344, F = 5.495, p-value = 0.012 | (3) |
| SOBS = –924.424 + 543.719 × pH + 1.962 × EC | r^2^ = 0.769, F = 34.859, p-value < 0.001 | (4) |
| Shannon = 2.963 + 0.004 × EC + 0.479 × pH | r^2^ = 0.338, F = 5.357, p-value = 0.013 | (5) |
| Chao = –1432.642 + 793.366 × pH + 2.232 × EC – 832.455 × P | r^2^ = 0.771, F = 22.501, p-value < 0.001 | (6) |
| ACE = –1601.525 + 830.783 × pH + 2.439 × EC – 892.694 × P | r^2^ = 0.790, F = 25.031, p-value < 0.001 | (7) |
| Simpson = 0.063 – 3.203 × organic matter + 0.075 × total nitrogen | r^2^ = 0.604, F = 16.021, p-value < 0.001 | (8) |
| N_2_-fixing = 0.004 + 0.001 × Bacteroidetes | r^2^ = 0.180, F = 4.843, p-value = 0.039 | (9) |
