# Supplementary material for: Agricultural management and cultivation period alter soil enzymatic activity and bacterial diversity in litchi (Litchi chinensis Sonn.) orchards
Source: Bot Stud. 2021 Sep 26;62:13. doi: 10.1186/s40529-021-00322-9 (PMC8473471; doi:10.1186/s40529-021-00322-9)
Supplement: Supplementary file 10 — Additional file 10: Table S8. Pearson correlation between temperature, relative humidity, and bacterial community. [file 40529_2021_322_MOESM10_ESM.docx]

**Table S8.** Pearson correlation between temperature, relative humidity, and bacterial community.

|  | SOBS | Chao | ACE | Shannon | Simpson |
| --- | --- | --- | --- | --- | --- |
| Temperature | 0.156 | 0.067 | 0.081 | **0.562**** | **0.679**** |
| Relative humidity | –0.055 | –0.003 | –0.004 | **–0.550**** | **–0.471**** |

Significance is indicated by **p-value < 0.01, and *p-value < 0.05.
