# Supplementary material for: Agricultural management and cultivation period alter soil enzymatic activity and bacterial diversity in litchi (Litchi chinensis Sonn.) orchards
Source: Bot Stud. 2021 Sep 26;62:13. doi: 10.1186/s40529-021-00322-9 (PMC8473471; doi:10.1186/s40529-021-00322-9)
Supplement: Supplementary file 11 — Additional file 11: Table S9. Pearson correlation between temperature, relative humidity, and enzymatic activity. [file 40529_2021_322_MOESM11_ESM.docx]

**Table S9.** Pearson correlation between temperature, relative humidity, and enzymatic activity.

|  | Acid phosphatase | Arylsulfatase | beta-glucosidase | urease | N-fixation |
| --- | --- | --- | --- | --- | --- |
| Temperature | **–0.359*** | **–0.489**** | –0.075 | **–0.704**** | 0.263 |
| Relative humidity | –0.040 | **0.818**** | –0.053 | 0.180 | 0.332 |

Significance is indicated by **p-value < 0.01, and *p-value < 0.05.
